# Current Applications and Future Perspectives of Fluorescence Light Energy Biomodulation in Veterinary Medicine

**DOI:** 10.3390/vetsci8020020

**Published:** 2021-01-25

**Authors:** Andrea Marchegiani, Andrea Spaterna, Matteo Cerquetella

**Affiliations:** School of Biosciences and Veterinary Medicine, University of Camerino, 62024 Matelica, Italy; andrea.spaterna@unicam.it (A.S.); matteo.cerquetella@unicam.it (M.C.)

**Keywords:** photobiomodulation, fluorescent light energy, canine dermatology, pyoderma, perianal fistula

## Abstract

The purpose of this review is to determine the state of the art of the mode of action and potential applications of fluorescence photobiomodulation in veterinary medicine. After a summary of the assets that have led the translation of such light-based therapies from bench side into clinical use, recent advances in canine dermatology using this brand-new approach are presented, and future scenarios where this type of care may provide benefits over the current standard care are highlighted.

## 1. Introduction

The use of therapeutic light and lasers has experienced an intense spread in the past few decades representing new appealing management options for dermatological clinical practice in present days [1]. In veterinary medicine, canine dermatological disorders account for the most commonly presented complaints in practice, being almost a quarter of all the case load [2]. These data suggest that despite considerable improvements in the prevention and therapy of some skin conditions, further efforts are needed to improve the management of many others. The skin represents the largest organ in the bodies of dogs and its problems are triggered not only by inadequate nutrition and/or hormonal imbalance; numerous agents including microorganisms, flea infestations, bacterial infections, physical or chemical agents, and immunological reactions are able to initiate and perpetuate dermatological disorders [2,3,4,5]. Being the outmost tissue in the body, the skin is easily accessible to a wide range of light-based treatments, which are characterized by unique features distinguishing them from most other therapeutic modalities, including: (I) ad hoc possibility to illuminate and treat selected areas, even at subcellular levels; (II) better treatment effects in terms of stimulation and inhibition of specific signaling pathways; (III) generally reduced risk of interference with other treatment modalities, prevention of systemic side effects, and very precise targeting of specific structures by exploiting their different light-absorbing properties [6]. For such reasons, the development and application of new therapeutic strategies in canine dermatology has attracted the interest of both researchers and trading companies. Different procedures involving the use of different light sources to interact with biological processes have been extensively studied and applied in human and veterinary medicine [6,7].

Low-level laser (light) therapy (LLLT), phototherapy, or photobiomodulation (PBM) are synonyms of the same practice that uses photons (mainly produced by light-emitting Diodes, LED) at different wavelengths and at non-thermal irradiance to influence biological activity [8]. LLLT has been successfully used in veterinary medicine in a wide variety of medical conditions including orthopedics [9], neurology [10], and wounds, as well as for many dermatological disorders such as hot spots (acute moist pyotraumatic dermatitis), otitis externa, licking granulomas [1], non-inflammatory alopecia [11], pododermatitis [12], and pruritus [13]. Up-to-date results of PBM in veterinary medicine have been extensively described elsewhere [14] and a number of studies have shown the beneficial effects of PBM, although the exact biochemical mechanism is not precisely known [8]. However, many studies have thrown light on the capacity of both laser and light therapy to alter the inflammatory response and isolated some of the cellular metabolites involved [6,7]. In fact, specific studies conducted both in vitro and in vivo have demonstrated that in PBM-treated tissues, there is an increase in reactive oxygen species (ROS) [15,16], adenosine triphosphate (ATP) [17,18,19], and nitric oxide (NO) [20]. The primary stage of such beneficial process is thought to be the photostimulation of enzyme cytochrome c oxidase in the mitochondrial respiratory chain [21]. Moreover, PBM is able to positively influence electron transfer and proton transportation, with a consequent increase in mitochondrial ATP production [22] increasing the amount of energy available for the cell to perform an array of processes, including healing [23]. 

More in general, PBM is able to influence several pathways. First of all, it is able to stimulate secretion of several growth factors including epidermal growth factor (EGF), vascular endothelial growth factor (VEGF), fibroblast growth factors (FGFs), transforming growth factor beta (TGF-β), and collagen (Coll). [24] PBM directly activates endogenous chromophores including cytochrome c oxidase [25], flavins [26], and opsins [27]. It can also modulate inflammation: both in vitro and in vivo studies have demonstrated the anti-inflammatory action of red (610–760 nm) light through the modulation of interleukin (IL)-1α and -β, IL-6, IL-17, and tissue necrosis factor (TNF)-α [28,29]. Therefore, as a consequence, PBM is responsible for the activation of fibroblasts, keratinocytes, and endothelial cells of exposed tissues thus producing a reduction of inflammation, pain, and edema [30]. Inflammation may also be modulated through matrix proteins such as matrix metalloproteinases (MMPs) and their tissue inhibitors [31]. 

## 2. Fluorescent Photobiomodulation and Its Mode of Action in the Healing Process

For all light-based treatments, light must be able to penetrate tissues in order to have any effect. Different devices for light-based treatments are available nowadays; they can be largely characterized based on whether they function primarily through heating (photothermal), mechanical perturbations (photomechanical), or chemical interactions (photochemical) [32]. The therapeutic effect of traditional lasers is based on the principle of selective thermolysis: the monochromatic and coherent light emitted at different wavelengths is absorbed by specific targets present within tissues (such as oxyhemoglobin, melanin, water, and others) which are selectively activated (or inactivated) [19]. Although thermal light radiation is usually emitted in a shorter time than the chromophore’s thermal emission and the procedure is considered safe, occasional and momentary side effects such as skin redness, itching, edema, appearance of small bruises, and slight burns have been described. In addition, precautions must be implemented to ensure safety of the patient and the practitioner when using lasers (especially class III and IV lasers) [33].

Research on PBM has led to the development of several light-generating devices and sources that can benefit a wide range of clinical indications. A novel approach to PBM is through application of a fluorescence light energy (FLE) system consisting of blue light which activates topical photoconverter hydrogel containing specialized chromophores (molecules able to be excited by certain wavelengths) that generate fluorescence [34,35]. Fluorescent (photo) biomodulation (FBM) uniquely uses chromophores embedded in a topical photoconverter substrate which can be found in different forms, such as, generally, silicone or nylon membranes (for human applications) or amorphous hydrogels, optimized for different therapeutic usage in both human and veterinary applications [34]. Irrespective of the material they are made of, the substrates themselves are not absorbed by the tissues, nor produce heat: instead, they are only employed to produce and deliver the fluorescent light energy to the tissue [36]. In addition, FLE is free from those side effects and risks related to the use of lasers.

In vitro studies were conducted to evaluate how FBM modulates cellular activity to improve human inflammatory skin conditions demonstrating a range of mechanisms. Among these, it has been shown that the production of inflammatory cytokines, TNF alpha and IL-6, produced by both human dermal fibroblasts (HDF) and epidermal keratinocytes in response to chronic inflammation, was decreased after FBM application [37,38].

The first application of FBM on canine spontaneous diseases was described by Scapagnini et al. in 2019 [34]. To ascertain both the effectiveness and effect of FBM on canine patients, fourteen dogs affected by deep pyoderma in at least two lesional areas underwent oral antibiotic therapy plus FBM application only in one site, as the other(s) served as the control. FBM was applied in a roughly 2 mm layer of photoconverter gel applied directly to the site to be treated and then illuminated with an LED device that delivered noncoherent blue light with peak wavelength between 440 and 460 nm and a power density of between 55 and 129 mW/cm^2^ for 2 min at approximately 5 cm of maximum distance. After illumination, the gel was gently removed using sterile gauzes immersed in sterile saline solution. FBM was applied twice weekly until clinical resolution, defined as the disappearance of the initially present lesions. At enrolment and then at healing from deep pyoderma, skin biopsies were obtained from both areas of the same dogs in all patients. These samples were assessed by immunohistochemistry considering the following parameters: epidermal growth factor (EGF), fibroblast growth factors (FGFs), transforming growth factor beta (TGF-β), tumor necrosis factor alpha (TNF-α), collagen (Coll) I and III, Ki67, factor VIII (FVIII), and decorin (DCN). In addition, using the same samples, mitochondrial analysis was conducted on dermal fibroblasts using a transmission electron microscope. As a result of this pilot evaluation, Scapagnini et al. found that all lesions reached complete clinical resolution (defined as the disappearance of lesions and restoration of normal skin) irrespective of FBM application. Interestingly, skin from sites receiving FBM exhibited less damage and less inflammation, as well as complete re-epithelialization, in addition to strong neoangiogenesis and the presence of synthetic activities of the connective matrix when compared with control sites. 

From the immunohistochemical point of view, FBM downregulated the expression of TNF-α and upregulated EGF, FGFs, TGF-β, Coll I and III, Ki67, FVIII, and DCN in a statistically significant manner in all cases, as per Table 1.

Similar results were obtained when applying FBM to surgical wounds [39]. Salvaggio et al. applied FBM to portions (a half) of surgical incisional wounds, finding a more physiological deposition of collagen, complete restoration, and less inflammation of the epidermal layer in comparison with the half of the wound that was not exposed. In addition, immunohistochemistry revealed upregulated expression of FVIII, EGF, DCN, Coll III, and Ki67 in FBM-exposed tissues [39].

Furthermore, the work of Scapagnini et al. also confirmed the impact of PBM, and for the first time attested the effect of FBM on mitochondria [34]. In fact, FBM-treated lesions revealed a 10-fold increase in the size of mitochondria compared with control lesions (90.15% increase vs. 9.09% increase, respectively; *p* < 0.0001). Moreover, microscopically, mitochondria from FBM lesions appeared to be more elongated and ovoid, with clearly defined cristae if compared to the baseline. Furthermore, FBM was responsible for a substantial increase (89.31%) in the number of mitochondria from the baseline to the healing time compared with only a 12.09% increase in control lesions, considering the same timeframe (*p* < 0.0001). 

Considering these beneficial effects on the healing process obtained by Scapagnini et al. with deep pyoderma, other research groups applied FBM in different canine dermatological conditions described hereafter as per Table 2. 

## 3. FBM in Veterinary Clinical Practice

At first, FBM was preliminarily used to manage (successfully) superficial bacterial folliculitis in dogs without the need for antimicrobials and with and an excellent safety profile with no therapy-related side effects [40] using the same application pattern described by Scapagnini [28]. Similarly, the same results were obtained in an exploratory study in which FBM was applied in association with systemic antibiotics to manage canine deep pyoderma [41]. One of the most frustrating dermatological disease in dogs in which FBM has been tested is interdigital pyoderma. Canine interdigital pyoderma (CIP), also referred to as interdigital furunculosis or pododermatitis, represents a frequent chronic inflammatory skin problem affecting one or more feet of dogs, usually due to an underlying disease (i.e., atopic dermatitis, cutaneous adverse food reaction, ectoparasites, endocrine disease, foreign bodies, and conformational problems) that predisposes them to deep bacterial infection of the pedal skin [47,48,49,50]. The condition generally requires prolonged courses of antibiotics, eventually in association with topical or systemic anti-inflammatory drugs, such as glucocorticoids or cyclosporine, to manage the inflammatory reaction and heal the patient [50]. For these reasons, FBM has been successfully applied in the management of CIP [42]. Thirty-six dogs with interdigital lesions diagnosed as CIP were randomly assigned to receive either systemic oral antibiotics alone or antibiotics plus FBM application twice weekly until complete resolution of CIP, defined as total disappearance of the lesions present at enrolment. Weekly clinical evaluation of dogs highlighted that starting from week 3 to the end of the study, FBM dogs showed a statistically significant improvement (*p* < 0.001) in the time needed to achieve clinical resolution in comparison with control dogs. In fact, FBM-managed dogs healed in a mean time of 4.3 ± 2.2 weeks (median, 3.5 weeks), whereas the control group needed 10.4 ± 4.9 weeks (median, 10.0 weeks) to be considered healed. The incidence of recurrence of the disease in a previously affected area was also taken into account as an exploratory endpoint, accounting for only one case in each group. All dogs included in the study that had *Staphylococcus pseudintermedius* and/or *Staphylococcus aureus* and neutrophils within phagocytosed bacteria were chosen as an indicator of both inflammation and bacteriological severity of the condition [51,52]. Previously, other authors assessed the in vitro bactericidal effect of blue light PBM on both methicillin-susceptible *Staphylococcus pseudintermedius* (MSSP) and methicillin-resistant *S. pseudintermedius* (MRSP) showing no significant reduction of colony counts for either bacteria [53]. In contrast to this, FBM was responsible for a quicker resolution of CIP when administered in association with systemic antibiotics, suggesting a possible synergistic effect of this combination. The reason for such effect of FBM is still unclear as the specific mode of action of FBM has not yet been fully elucidated. FBM has also been successfully applied in human medicine in the treatment of both infectious and non-infectious inflammatory skin conditions [54] and it is very likely that the positive and beneficial effects seen in CIP dogs are related to such anti-inflammatory and antibacterial properties. As a limitation of the study, quality of life, which is deeply affected by such condition [55,56], was not taken into account despite representing a useful tool for assessing disease severity and treatment efficacy [57]. In addition, it would be of great interest to notice a possible antipruritic effect of FBM, which deserves to be investigated. PBM without FLE has been already used in dogs affected by pedal skin disease with contradictory results: a pilot study conducted on five dogs affected by sterile pyogranulomatous pododermatitis reported a beneficial effect of low-level laser therapy [12] while, on the other hand, a larger study revealed no significant improvement of pedal pruritus in atopic dogs [13]. In contrast, FBM contributed to an accelerated clinical resolution of CIP cases when administered with systemic antibiotics, performing better than PBM [42]. 

After the favorable results in CIP, FBM has been successfully applied to manage bacterial skin infections associated with canine calcinosis cutis lesions [58]. A 15-year-old male Golden Retriever presenting multifocal symmetrical alopecia, skin nodules, and plaques on the dorsal part of the body was diagnosed with calcinosis cutis due to suspected iatrogenic hypercortisolism due to prednisolone administration over the previous two years. FBM was applied weekly to manage bacterial skin infection as an addition to systemic antimicrobial and topical (spray and shampoo) therapies. To assess a possible beneficial effect of FBM, part of the lesion was protected from FBM to not receive the treatment and served as the control. During treatment, the skin improved and fewer comedones were visible and present, especially in the FBM-treated areas. After three weeks of FBM, the dog’s hair started to slowly regrow and, at week 4, pyoderma was improved enough to discontinue systemic antibiotic treatment and keep the patient under topical therapy alone, reducing the need for further antibiotic administration. After three additional weeks, pyoderma completely resolved and FBM was discontinued. Cytology was conducted weekly during treatment and confirmed accelerated improvement of FBM-managed lesions compared with unexposed ones. In addition, FBM areas had no bacterial infection, whereas other areas were still infected, confirming a possible antibacterial effect of FBM [58]. Even if this is only a case report, FBM was able to control bacterial overgrowth in this complicated case. 

Remaining in the skin district, FBM has been successfully used to manage cutaneous wounds in elderly patients [59]. Traumatic wounds represent a fairly common reason for the admission of dogs to a veterinary practice and several factors have to be taken into account for the choice of a treatment protocol, such as time delay between injury and treatment, contamination, extension and depth of the wound, clinical status and age of the patient [60,61,62]. In elderly patients, wound management can be challenging due to both the concurrent diseases and impaired physiological state, potentially resulting in delayed healing and chronic or non-healing wounds [63], making wound dressing one of the most often applied approach [64]. Two elderly dogs presented with similar wounds at the neck level characterized by significant loss of substance in the dorsal region. Both dogs underwent surgical debridement and both anti-inflammatory and antimicrobial therapy was commenced in both dogs; the administration of drugs was discontinued after two and three weeks, respectively. In addition, FBM was applied once weekly, with two cycles of illumination in the same session (2 min plus 2 additional minutes, “back-to-back” approach) until complete restoration of the skin barrier was achieved in 9 and 16 weeks, respectively. This case report provides the first evidence that FBM may be able to provide an ideal environment for second intention healing of wounds in elderly dogs, setting the basis for further clinical investigation needed to better characterize the therapeutic potential of FBM application in veterinary wound healing. The bias of the present study is represented by initial antibiotic and anti-inflammatory therapy received by both dogs, which could have exerted some facilitative effect in the reparative process. Further clinical controlled and randomized studies are needed to ascertain the therapeutic potential of FBM application in wound healing, particularly in comparison with other wound management modalities. 

Another stimulating application of FBM was to canine perianal fistulas (CPF) [45]. CPF, also referred to as anal furunculosis, is characterized by the presence of painful and chronic inflammatory sinus tracts and ulcers in the perianal skin of dogs [65]. The most commonly affected breed is German Shepherds, suggesting a potential genetic component to disease susceptibility in this breed [66]. An immune-mediated pathogenesis for CPF has been ascertained [67,68,69] and, despite different treatment modalities being available (medical, surgical, or a combination of both), euthanasia is still required by some owners [65]. FBM has been successfully applied in four German Shepherds to manage and resolve fistulization and discomfort during CPF [45]. Dogs presented with clinical signs consistent with different severity levels of CPF. Prior to presentation, all of them had been kept under antimicrobials and/or immunomodulatory therapies (ciclosporin or prednisolone), with weak to mild improvements. At the time of FBM application, antimicrobials and immunomodulatory drugs were discontinued for at least 14 and 30 days, respectively. FBM, as for the cutaneous wound cases described above, was applied weekly with two consecutive applications in the same session and no concomitant medications (neither antimicrobials nor immunosuppressants) were administered. All dogs registered a marked improvement related to FBM: after four weeks, lesional areas decreased by close to 75% on average and at week 6, three out of four dogs were considered healed enough to stop FBM. Only one dog required more than seven weeks to stop FBM and no recurrence was observed by owners within the first six months after cessation of light therapy. Noteworthily, FBM was also responsible for rapid and long-lasting improvement in non-dermatological signs such as tenesmus, hematochezia, malodorous perianal area, pain, discomfort, licking, and inability to sit. Such results obtained in this case series advice the potential of FBM for CPF cases, representing an effective and owner-compliant alternative to conventional therapy, especially considering that dogs received FBM and hypoallergenic diet as the only management tool. It would be of interest to have more data from CPF-affected dogs regarding a possible presence of colitis, as it has been speculated that in humans perianal furunculosis could be linked to colitis or even Chron’s disease, for which German Shepherds could represent a spontaneous model to evaluate and use at the preclinical stage for such desirable application [70,71]. 

Notwithstanding the favorable outcomes, the study presents some limitations in the size of the sample (too small), scoring system (recognized and validated system is absent), as well as the quality of life of both dogs and owners that was not taken into account and evaluated. In addition, for that dog that required more than seven weeks of FBM, a skin biopsy of the perianal area could have been taken to evaluate possible concomitant conditions. Despite these facts, this small exploratory study suggests that FBM may be a useful management tool for dogs affected by CPF and deserves to be further and deeply investigated, especially in terms of its mode of action in such immune-mediated diseases.

The efficacy of FBM has also been assessed preliminarily in the course of spontaneous otitis in dogs [46]. Canine otitis externa is an uncomfortable condition that is challenging to manage because owner compliance tends to decrease with time and some dogs may display discomfort or aggressiveness when an attempt to administer in-ear products is made due to a sore ear canal [72,73]. FBM was applied either once or twice weekly (for a total of six applications) in comparison with enrofloxacin plus silver sulfadiazine administered twice daily for three weeks. While all treatments showed an ameliorative effect on the severity of otitis, FBM applied twice weekly was responsible for the most favorable results in terms of the OTIS-3 index scoring system, pruritus, and pain, as well as cytological and bacteriological assessments. These results are even more impressive if one takes into account the fact that the OTIS-3 score and some of the other parameters considered were statistically significantly higher at enrolment in the FBM twice weekly group than in the others [46]. One of the biases of the study is the difference at enrolment in OTIS-3 scores and in some of the secondary parameters measured between groups. Another limitation is the fact that cytological and microbiological assessments were performed blindly, but authors did not guarantee maintenance of blindness throughout the study. Although it is possible to hypothesize that further research is needed prior to having a final device for the application of FBM in canine otitis, the results presented in the abovementioned study highlight a beneficial anti-inflammatory and antiseptic effect of the treatment in this field [46], possibly supporting the healing process without burden on owner compliance. 

## 4. Conclusions

FBM is a treatment modality that employs fluorescent light energy to interact with tissues and it has been extensively applied in clinical dermatology for the treatment of acne [74] and chronic non-healing wounds such as venous leg ulcers (VLUs), diabetic foot ulcers (DFUs), and pressure ulcers (PUs) [36,75,76,77]. 

The studies described in this literature review are the evidence that PBM can exhibit its aptitudes in veterinary medicine. Being able to reduce inflammation and control bacterial overgrowth, it has the potential to be considered as an option in different dermatological conditions, possibly replacing some topical treatments and improving owner compliance.

FBM may be an effective sole treatment for canine superficial pyoderma, eliminating the need for, or decreasing the length of time of administering systemic antibiotics and supporting antimicrobial stewardship programs [78,79,80,81,82,83]. In addition, it has the potential to accelerate time to clinical resolution for CIP compared with systemic antibiotic treatment and may represent a valid option for those cases of canine perianal fistulas. Systemic antibiotic therapy for canine pyodermas is becoming more problematic because of the increasing incidence of antimicrobial resistance and methicillin-resistant *Staphylococcus* (MRS) [84,85]. To help address this issue, topical therapies, either as monotherapy or as part of a multitherapeutic approach, are becoming an essential component of the management of dermatological condition, with the aim to decrease the length of time administering or eliminate the need for systemic antibiotics and to prevent persistent carriage of methicillin-resistant *Staphylococcus aureus* (MRSA) in human beings [86,87].

FBM also supports owner compliance as much as possible, which is critical to the resolution of pyodermas and prevention of recurrence. FBM supports owners as much as possible to promote effective compliance and relieve them of administration of home therapies.

Further studies are needed to better ascertain the potential of FBM in veterinary medicine and possibly find other indications for its application, especially in feline dermatology. The future of FBM in veterinary medicine is embedded in the challenging panorama of a rapidly developing world, but if this challenge could be overcome, the resultant comprehensive treatment systems could address multiple pathologies, possibly without relying on pharmaceuticals, which could be a significant improvement over the piecemeal application approaches that are currently employed. 

The technologies behind light-based therapies are advancing rapidly, and in many cases, their utility in the clinical setting is still being actively explored. It is realistic to hypothesize that, as this technology can be optimized for dermatological applications, its value in clinical veterinary dermatological care will continue to grow.

## Figures and Tables

**Table 1:** Overview of cytokines influenced by PBM and FBM.

Cytokines	Regulation
EGF	↑
FGF	↑
TGF-β	↑
TNF-α	↓
Coll I	↑
Coll III	↑
Ki67	↑
FVIII	↑
DCN	↑

**Table 2:** Overview of clinical FBM applications in canine patients.

Disorder	References
Superficial bacterial folliculitis	[40]
Deep pyoderma	[41]
Interdigital furunculosis	[42]
Wounds	[43,44]
Canine perianal fistulas	[45]
Otitis	[46]

## Data Availability

No new data were created or analyzed in this study. Data sharing is not applicable to this article.
